# Cases of Hydrocephalus Successfully Treated by Iodide of Potassium

**Published:** 1849-08

**Authors:** William Gardener

**Affiliations:** Philadelphia


ART. IV.—*Cases of Hydrocephalus successfully treated with Iodide of Potassium, by* William Gardner, M. D., (read before the Medico Chirurgical College of Philadelphia.)

The following cases of Hydrocephalus lately came under my notice, and were successfully treated with the Hydriodate of Potash:—*Case* 1. J. T., æt four months, was unusually fretful and restless for about two weeks before any particular notice was taken of it, under the impression the child was teething, when he was taken suddenly worse, and I was requested to attend him. Found him in a very restless state, constantly rolling his head from side to side; frequent vomiting; bowels costive; great heat in head; contracted pupils, &c., in fact, a well marked case of Hydrocephalus. Leeches were applied to the temples, cold to the head, mustard plasters over the stomach and along the spine, bowels moved by injection, and castor oil by the mouth, and small doses of calomel and ipecac every two hours. Leeching occasionally, calomel and ipecac with potassæ nit. and digitalis, the warm bath, sinapisms to the spine and extremities, constituted the treatment for several days, but without any benefit, the case, to all appearances, rapidly hastening to a fatal close. Effusion took place, and he lay in convulsions several hours, and for two days refused the breast. Upon the supervention of effusion I gave him the following mixture,—℞. Iodidi Potassii ʒj, Syr. Prun. Virg. ʒij, Aquæ ʒij, teaspoonful every two hours, with the happiest result. He gradually recovered consciousness, and, in about two weeks from the commencement of the Iodide, was perfectly recovered, and has continued well since. His head was carefully measured with a tape on the morning he commenced taking the Iodide, and was found to measure full an inch larger than after his recovery.

*Case* 2. J. R., aged two years, while playing on Sunday evening, Jan. 1849, fell with considerable force on the back of his head, which stunned him for some minutes. He was soon after put to bed, and slept soundly all night. The following morning he vomited and refused his breakfast, was fretful and feverish. A physician saw him during the day, and attended him until the following Friday, when I first saw him, and he had then considerable heat about the head, strong active pulse, great restlessness, starting and screaming, contracted pupils, &c. Directed a warm bath, mustard plasters to feet, the head to be kept cold with iced water, leeches to the temples, and cal. and ipec. with pot. nit. every two hours. Saturday, much the same; leeches had not been applied owing to a prejudice of the friends. Continue treatment. Sunday evening, considerably worse; twelve leeches were applied to the temples with relief. ℞. Calomel and Ipecac. a.a. gr. ss. Digitalis gr. $\frac{1}{8}$, Potas. Nit. gr. vj, every two hours. Monday, effusion evidently taken place, laying on his back in a state of stupor, pupils dilated, paralysis of the right side. Gave him ℞. Potassii Iodidi ʒss, Syr. Prun. Virg. ʒij, Aqua. ʒij; teaspoonful every two hours, and the following powders every alternate hour:--℞. Calomel, Pulv. Ipecac. a.a. gr. $\frac{1}{2}$, Pulv. Acaciæ gr. j. Tuesday, slight improvement, continue mixture and powders, apply mustard plasters occasionally the whole length of spine, and to the extremities. Wednesday, considerably better, consciousness returning gums slightly affected. Omit the powders, continue the mixture. He took the mixture till the following Monday, when there was apparently nothing the matter with him but weakness.

Philadelphia, June, 1849.

